# Case of Delirium Tremens Treated by Inhalation of Ether, at the Seamen’s Retreat, Staten Island

**Published:** 1847-12

**Authors:** Wm. C. Anderson

**Affiliations:** Resident Physician


*Case of Delirium Tremens Treated by Inhalation of Ether, at the Seamen's Retreat, Staten Island.* By Wm. C. Anderson, M.D., Resident Physician.—While the subject of the inhalation of sulphuric ether is attracting the attention of the profession, and much has already been adduced in favor of this new agent as a means of preventing pain in surgical operations—in fact, of divesting the knife of nearly all its terrors, and even parturition of its pangs—very little is known of its powers as a remedial agent.

For this reason, the following case may not be considered as devoid of interest:

J. Miller, æt. 53, seaman, attached to the United States ship Savannah, admitted to the hospital October 4th; was paid off about two weeks ago, and has not been sober since. During the last two days had made several attempts at self-destruction; on admission, quite delirious, and so violent that it was necessary to confine him by strapping him to the bedstead. Pulse 104, rather feeble; tongue coated; pupils natural. ℞. Inf. senna comp. ℥iv.

Oct. 5th. Talking and singing all night; bowels not moved. Repeat purgation; lemonade to drink; diet, arrowroot and milk.

5, p. m. Same condition. Continue treatment. ℞. Tr.

opii ʒss, tr. humuli lup. ℥iij. M. Dose, a table-spoonful every hour.

Oct. 6th. Condition the same; no sleep; pulse 120. ℞. Emp. vesic. to nape; head shaved; apply ice. Bowels being still confined, administered an enema by means of the long tube; this operated freely. Continue opiate mixture.

9, p. m. Still continues without sleep, and continually raving.

Oct. 7th. Delirium continued all night without intermission; pulse weaker.

12 o'clock, m. Skin cold; pupils contracted. ℞. Milk punch; omit ice; soup diet.

10 minutes past 9, p. m. His condition is altered for the worse; body bathed in a cold sweat, which about the head and face is quite profuse; pupils contracted to the size of pin-holes; refuses to take his medicine or drinks, which, when put into his mouth by force, he spits out again, without swallowing a particle.

Such being the condition of things, it was decided to administer the ether, which was done by means of a hollow sponge, supplied from the outside, and covering the whole face. At first he resisted; but although there was no possibility of making him swallow medicines, there was no resisting inhalation. After four minutes' application, he became perfectly passive, and commenced drawing long and deep inspirations, with stertor and tracheal rale; pupils still contracted, and balls turned upwards. The sponge was then removed during five minutes, etherization continuing during that time, when it gradually passed off, the breathing becoming more natural, and a return of restlessness and delirium took place. The sponge was reapplied, and after four or five minutes the effects again became manifest, and continued for from eight to ten minutes; and when they passed off, he remained much more calm, although not intelligent nor seemingly inclined to sleep. Another application was made, with the same results; he no longer resisted the inhalation, but inspired with eagerness when the sponge was applied, and after recovering from the stupor, did not refuse his drinks. He dozed occasionally for four hours; took one pill of morphine, gr. ss., which had been left with the attendants to be given him if he did not sleep, and fell asleep shortly after, and did not awake for six hours, when he was found to be perfectly rational, saying that he felt himself quite well.

This case, when the ether was resorted to, was certainly a most unpromising one; but whether the induction of sleep must be attributed to this agent, or the narcotics previously taken, or the pill of morphine taken afterwards, may admit of doubt; but that the patient was quieted, and from being violent, resisting and furious, was rendered quiescent, passive and obedient, there can be no question.—*New York Annalist.*

